# Microenvironmental changes during differentiation of mesenchymal stem cells towards chondrocytes

**DOI:** 10.1186/ar2153

**Published:** 2007-03-29

**Authors:** Farida Djouad, Bruno Delorme, Marielle Maurice, Claire Bony, Florence Apparailly, Pascale Louis-Plence, François Canovas, Pierre Charbord, Danièle Noël, Christian Jorgensen

**Affiliations:** 1 Inserm, U 844, 80 avenue Augustin Fliche, Montpellier, F-34091 France; 2 Université Montpellier 1, 2 rue Ecole de Médecine, Montpellier, F-34000 France; 3 Inserm, ESPRI EA3855, 10 bld Tonnellé, Tours, F-37032 France; 4 Genopoietic, 1390 rue Centrale, Beynost-Miribel, F-01708 France; 5 CHU Montpellier, Hôpital Lapeyronie, avenue du Doyen Gaston Giraud, Montpellier, F-34295 France

## Abstract

Chondrogenesis is a process involving stem-cell differentiation through the coordinated effects of growth/differentiation factors and extracellular matrix (ECM) components. Recently, mesenchymal stem cells (MSCs) were found within the cartilage, which constitutes a specific niche composed of ECM proteins with unique features. Therefore, we hypothesized that the induction of MSC differentiation towards chondrocytes might be induced and/or influenced by molecules from the microenvironment. Using microarray analysis, we previously identified genes that are regulated during MSC differentiation towards chondrocytes. In this study, we wanted to precisely assess the differential expression of genes associated with the microenvironment using a large-scale real-time PCR assay, according to the simultaneous detection of up to 384 mRNAs in one sample. Chondrogenesis of bone-marrow-derived human MSCs was induced by culture in micropellet for various periods of time. Total RNA was extracted and submitted to quantitative RT-PCR. We identified molecules already known to be involved in attachment and cell migration, including syndecans, glypicans, gelsolin, decorin, fibronectin, and type II, IX and XI collagens. Importantly, we detected the expression of molecules that were not previously associated with MSCs or chondrocytes, namely metalloproteases (MMP-7 and MMP-28), molecules of the connective tissue growth factor (CTGF); *cef10/cyr61 *and *nov *(CCN) family (CCN3 and CCN4), chemokines and their receptors chemokine CXC motif ligand (CXCL1), Fms-related tyrosine kinase 3 ligand (FlT3L), chemokine CC motif receptor (CCR3 and CCR4), molecules with A Disintegrin And Metalloproteinase domain (ADAM8, ADAM9, ADAM19, ADAM23, A Disintegrin And Metalloproteinase with thrombospondin type 1 motif ADAMTS-4 and ADAMTS-5), cadherins (4 and 13) and integrins (α4, α7 and β5). Our data suggest that crosstalk between ECM components of the microenvironment and MSCs within the cartilage is responsible for the differentiation of MSCs into chondrocytes.

## Introduction

In articular cartilage, chondrocytes were thought to represent a unique cell type, but with a phenotype differing in the superficial, mid, deep and calcified zones [1]. However, mesenchymal stem cells (MSCs) have been recently identified in articular cartilage and are thought to represent up to 3.5% of the constituent cells [2]. The number of MSCs might even increase in the cartilage of patients with osteoarthritis (OA), compared with healthy cartilage, raising the possibility that these progenitor cells would be involved in the pathogenesis of arthritis, differentiating abnormally in response to the inflammatory milieu of the joint and signals from the extracellular matrix (ECM). The role of MSCs present in the cartilage is unknown.

Adult MSCs are pluripotent progenitor/stem cells; their progeny includes chondrocytes, tendon cells, haematopoiesis-support stromal cells, adipocytes and osteoblasts [3,4]. MSCs, similar to other stem cells, have an essential role in the regeneration/maintenance of the adult tissues submitted to physiological modelling/turnover or following injury. The fundamental property shared by all stem cells is their ability to balance the cell-fate decision between self-renewal and differentiation. The microenvironment regulates the maintenance of the stem-cell pool and commitment towards specific lineages through intrinsic and extrinsic factors, creating niches. For this regulation, adhesion of stem cells to the ECM is crucial. Cells and ECM adhesion molecules that enable cell communication are the prerequisite for tissue formation and maintenance.

The mechanisms that regulate chondrogenic differentiation include both autonomous (stem-cell intrinsic) and non-cell-autonomous (microenvironmental) components. Chondrogenesis is driven by a coordinated effect of hormones (such as parathyroid hormone (PTH)), morphogens (such as Hedgehog (Hg) or wingless (Wnt) proteins) and cytokines (such as members of the bone morphogenetic protein (BMP) and transforming growth factor (TGF)-β family) through their respective receptors [5]. However, many other factors drive the differentiation of MSCs towards cartilage, including ECM molecules, such as proteoglycans (PGs; syndecans and glypicans) or fibulins [6,7]. Members of the connective tissue growth factor (CTGF); *cef10/cyr61 *and *nov *(CCN) family, in addition to molecules with A Disintegrin And Metalloprotease domain (ADAM), and integrins have also been shown to have a crucial role in chondrogenesis [8]. These ECM molecules might interact with growth factors, chemokines or members of the Wnt family, or their receptors, to modulate their signalling [9]. Studies have demonstrated that normal chondrocytes adhere to various amounts of type I and IV collagens, thrombospondin, vitronectin, fibronectin, laminin and fibrinogen through the RGD (Arg-Gly-Asp) sequence and integrin-mediated interactions [10]. Indeed, there is a vast range of cellular responses to cell–matrix interactions, depending on the integrin receptors expressed by the cell and the composition of the surrounding ECM.

Because the cartilaginous microenvironment is composed of ECM proteins, closely associated to stem cells and chondrocytes, we hypothesized that the molecules of the ECM might create a niche specifying chondrocytic differentiation of MSCs *in situ *and, therefore, that the corresponding receptors would be differentially expressed in the undifferentiated MSCs compared with fully differentiated chondrocytes. We previously established a genomic profile of human MSCs before and after their differentiation into chondrocytes, using the cDNA chip technology (F Djouad, D Noël, unpublished data). However, this technology is currently limited by the relative lack of reproducibility, absence of quantitative results and amounts of required RNA. To elucidate the microenvironmental signals involved in the chondrogenic differentiation of MSCs, we designed a large-scale Taqman^® ^low-density array (TLDA) (Applied Biosystems, Courtaboeuf, France) using real-time RT-PCR, enabling the simultaneous quantitative analysis of 384 mRNA transcripts. The data have been assembled into a biological process-oriented database, serving as a model for the cartilage MSC niche.

## Materials and methods

### Cell culture

Human MSC cultures were established from bone-marrow aspirates of two healthy donors (aged 36 and 40 years) after informed consent. Mononuclear cells were plated at a density of 5 × 10^4 ^cells/cm^2 ^in α-minimum essential medium (α-MEM), supplemented with 10% fetal bovine serum (FBS; Perbio Science France SAS, Brebières, France), 1 ng/ml basic fibroblast growth factor (b-FGF), 100 U/ml penicillin and 100 μg/ml streptomycin. When cultures reached near confluence, cells were detached with 0.05% trypsin and 0.53 mM ethylene diamine tetracetic acid (EDTA) and subsequently re-plated at a density of 1,000 cells/cm^2^. MSCs were used at passage 3 to 4 and shown to be positive for CD44, CD73, CD90 and CD105 and negative for CD14, CD34 and CD45, as previously described [11].

### *In vitro *chondrogenic differentiation

Chondrogenic differentiation of MSCs was induced by 21-day culture in micropellet [12]. Briefly, MSCs (2.5 × 10^5 ^cells) were pelleted by centrifugation in 15 ml conical tubes and cultured in BMP-2-conditioned chondrogenic medium. Conditioned medium consisted of the supernatant of C9 cells cultured for 48 hours in DMEM supplemented with 0.1 μM dexamethasone, 0.17 mM ascorbic acid and 1% insulin-transferrin-selenic acid (ITS) supplement (Sigma, l'Isle d'Abeau, France) [12]. C9 cells derived from the C3H10T1/2 murine MSC line expressing human BMP-2 (1,231 ng/24 h/10^6 ^cells) under control of a TetOff promoter [13]. As a control, conditioned media from C3H10T1/2 cells were unable to induce any cell differentiation, at least for the chondrogenic- and osteogenic-specific markers tested with semiquantitative RT-PCR (data not shown).

### RNA preparation

At day 0, MSCs cultured in monolayer were harvested by treatment with 0.05% trypsin and 0.53 mM EDTA, washed with PBS and pelleted at 300 g for 5 minutes at 4°C. At day 2, 7 and 21 of chondrogenesis, micropellets (15 to 20) were recovered, washed in PBS and mechanically dissociated. Total RNA (3 μg) was extracted using the RNeasy (Quiagen S.A., Courtaboeuf, France) kit, according to the recommendations of the manufacturer.

### TaqMan^® ^real-time RT-PCR

TLDAs (microfluidic cards; Applied Biosystems) were used in a two-step RT-PCR process [14]. First-strand cDNA was synthesized from 3 μg total RNA using the high-capacity cDNA archive kit (Applied Biosystems). Quantitative PCR reactions were then carried out using the microfluidic cards and the ABI PRISM 7900HT Sequence Detection System (Applied Biosystems). The 384 wells of each card were preloaded with predesigned fluorogenic TaqMan^® ^probes and primers. cDNA (800 ng) combined with 1X TaqMan^® ^Universal Master Mix (Applied Biosystems) were loaded into each well (2 ng/well). The microfluidic cards were thermal cycled at 50°C for 2 minutes and 94.5°C for 10 minutes, followed by 40 cycles at 97°C for 30 seconds and 59.7°C for 1 minute. Data were collected using instrument spectral compensations by the SDS 2.1 software (Applied Biosystems) and analysed using the threshold-cycle (Ct) relative-quantification method. The content of the cDNA samples was normalized by subtracting the number of copies of the endogenous glyceraldehyde-3-phosphate dehydrogenase (GAPDH) reference gene from the Ct of the target gene (ΔCt = Ct of target gene - Ct of GAPDH). The results for the complete list of the tested transcripts are expressed as the mean of 2^-ΔCt ^± the standard error of the mean (SEM) at the different time points and shown as supplementary data (Additional file 1).

### Fluorescence-activated cell sorter analysis

MSCs were plated in tissue-culture flasks in chondrogenic medium, with or without 10 ng/ml hBMP-2 (R&D Systems, Lille, France), and cultured for 48 hours. Cells were harvested by treatment with 0.05% trypsin and 0.53 mM EDTA. After chondrogenic induction, micropellets were dissociated by treatment with 2 mg/ml collagenase type IA-S (Sigma). After a wash with PBS, isolated cells were suspended in PBS containing 0.1% BSA and 0.01% sodium azide and incubated on ice with primary mAbs for 30 minutes. The mouse mAbs used were specific for human CD29 (integrin β1, cloned March 4), CD49a (integrin α1), CD49d (integrin α4), CD49e (integrin α5), CD49f (integrin α6), CD106 (vascular cell adhesion molecule; VCAM1), CD146 (melanoma cell adhesion molecule; MCAM), chemokine CC motif receptor (CCR)3, CCR4, chemokine CXC motif receptor (CXCR)4 or isotypic controls (R&D Systems). Flow cytometry was performed on a fluorescence-activated cell sorter (FACS) scan and data were analysed with the Cellquest software (BD Pharmingen, Le Pont de Claix, France). Data are expressed as the percentage of cells positive for the marker analysed ± SEM.

## Results and discussion

Chondrogenesis is tightly regulated by growth and differentiation factors, involving prominently the FGF, TGF-β, BMP, Wnt and Hg pathways [14]. This process is controlled by cellular interactions with the surrounding matrix and other environmental factors that initiate or suppress cellular signalling in a spatiotemporal manner, including the level of oxygen, mechanical tension and cellular contact with the components of the cartilaginous matrix, which are essential for the maintenance of the adult tissue homeostasis [5]. Using the microarray technology, we previously investigated the gene-expression profile of human bone-marrow-derived MSCs for 21 days, before and after their differentiation into chondrocytes, using the micropellet culture system and hBMP-2 as the inducing factor (F Djouad, D Noël, unpublished data). Of the genes upregulated during chondrogenesis, numerous genes corresponding to constituents of the ECM or membrane-bound proteins were identified. We thus investigated whether such genes might be modulated during the time-course of chondrogenesis and take part in the differentiation process. We took advantage of using the TLDA, which enables precise and simultaneous quantification of the expression of 384 different mRNAs in a single experiment. We designed a card with primer sets corresponding to genes belonging to the stem cell, osteoblast, chondrocyte, adipocyte and myocyte signatures. Of the 384 mRNAs tested, 16% were not expressed at any time and 21% are presented below (Additional file 1).

### Time-course expression of chondrocyte-specific markers during the differentiation of mesenchymal stem cells

**Figure 1:**
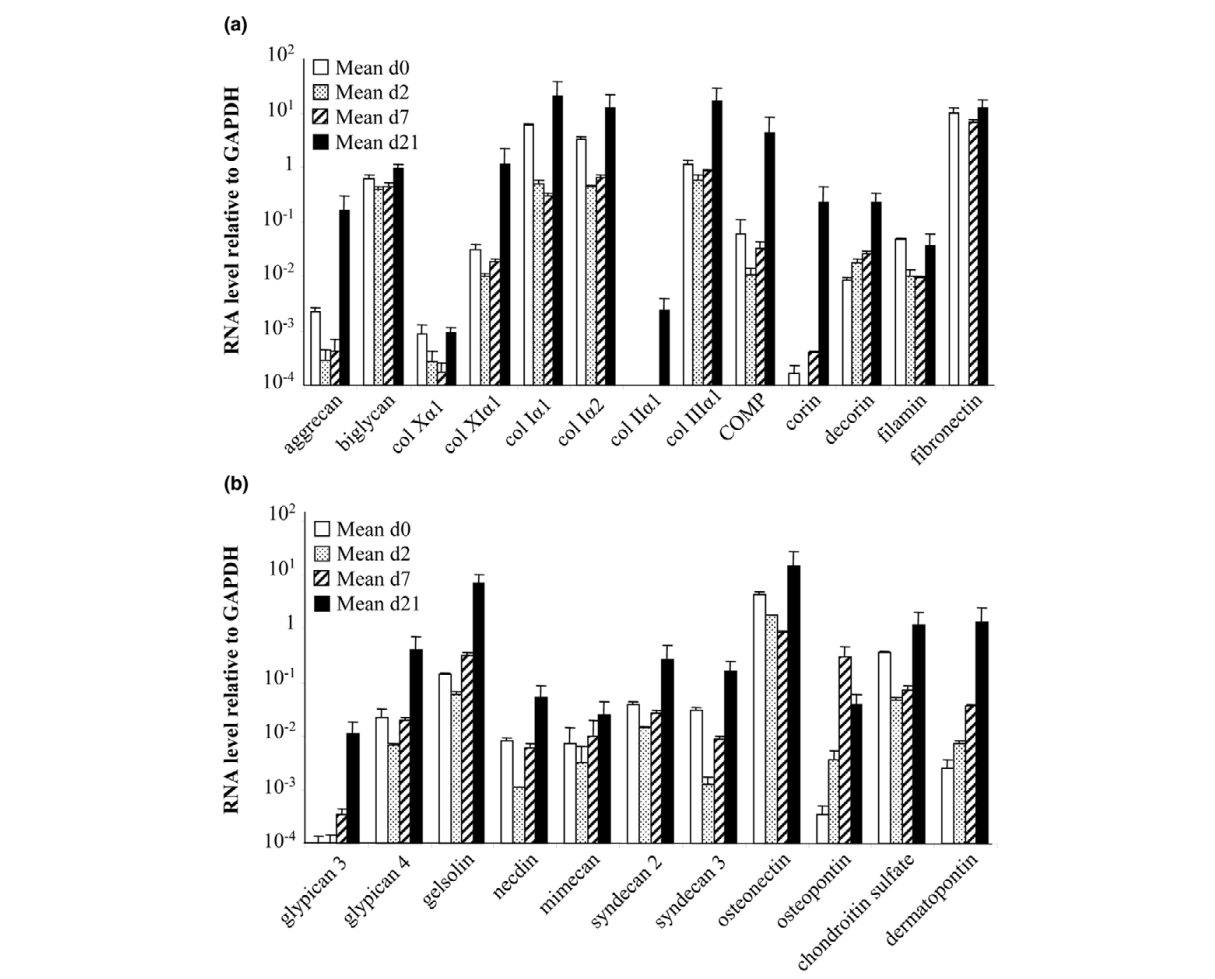
Time-course expression of major components of the chondrocyte-associated extracellular matrix (ECM). The gene-expression profile of mesenchymal stem cells (MSCs; *n *= 2) was analysed by real-time PCR during their differentiation towards chondrocytes in micropellets. **(a) **and **(b) **Change in the expression levels of various proteoglycans (PGs), collagens and proteins of the ECM. Genes were arbitrarily distributed. COMP, cartilage oligomeric matrix protein; GAPDH, glyceraldehyde-3-phosphate dehydrogenase; PCR, polymerase chain reaction.

First, we confirmed that MSCs underwent chondrogenesis by determining the expression of the mRNA specific for aggrecan and type II collagen at day 21 by quantitative RT-PCR (data not shown). Second, we performed the TLDA on RNA extracted at different time points during chondrogenesis. In cartilage, there are several different types of collagens and PGs. PGs can be divided into the following types: ECM-associated components, such as aggrecan, the major chondroitin sulphate PG, in addition to small leucin-rich PGs, such as decorin and biglycan; and cell-surface components, the glypicans and syndecans. Of the cell-surface components, syndecan-4 is more abundant than syndecan-2, whereas syndecan-3 is briefly expressed during the early stages of chondrogenesis [15]. Through their heparin and chondroitin sulfate PGs, both glypicans and syndecans interact with growth factors, such as FGF, Hg and Wnt, and their receptors, affecting their biological effects by modulation of their signalling. Although their role has been controversial, the binding of ligands to the heparan sulfate PGs might protect the ligand from the receptor, giving it a change to diffuse over a longer period of time [16] and participate in the regulation of the cellular phenotype within the cartilage. In this study, the expression kinetic of various ECM transcripts shows that, at the early stage of the differentiation process (day 2 and 7), most of the transcripts were downregulated, whereas they were upregulated by more than threefold on day 21. Of these transcripts, only type II collagen and glypican 3 were absent in MSCs, whereas all of the other transcripts were expressed at various levels on day 0 (Figure 1a,b). The data confirmed the upregulation of PGs and collagens known to be overexpressed and/or specific to the articular cartilage, such as aggrecan, biglycan, glypicans, mimecan, syndecans, decorin, and type II, III, X and XI collagens (Figure 1a,b). The cartilage also contains numerous proteins that are neither collagens nor PGs and have a structural role in the matrix, such as cartilage oligomeric matrix protein (COMP), dermatopontin and fibronectin, or are part of the cytoskeleton, such as filamin [17]. Other proteins, such as osteonectin and osteopontin, are involved in the mineralization of cartilage. The corresponding mRNAs were increased on day 21, except for filamin (Figure 1a,b).

Gelsolin is involved in the cytoskeletal organization and induced by integrin signalling. The expression of gelsolin and mimecan was recently described in OA chondrocytes, in addition to the upregulation of gelsolin in hypertrophic chondrocytes [18,19], but, to our knowledge, the expression of gelsolin in MSCs has not been previously reported. Of the proteins that have not been reported to have a role in cartilage, corin and necdin are of interest. The expression of corin in MSCs and its upregulation in chondrocytes was unexpected because this protein has been associated with hypertrophic cardiomyocyte and myocardium failure [20]. Necdin is an inductor of myogenesis [21] and inhibitor of brown adipogenesis [22]. However, the increased expression of these two proteins during chondrogenesis (1,370- and 6.5-fold, respectively) might reflect an important role during MSC differentiation.

In summary, the upregulation of these various molecules known to be associated with the chondrocyte phenotype confirms that chondrogenic differentiation occurs under our experimental conditions and validates the use of the quantitative PCR assay for the detection of various ECM or membrane-associated components and the analysis of their potential role in chondrogenesis.

### Modulation of the expression of secreted proteins during chondrogenic differentiation

**Figure 2:**
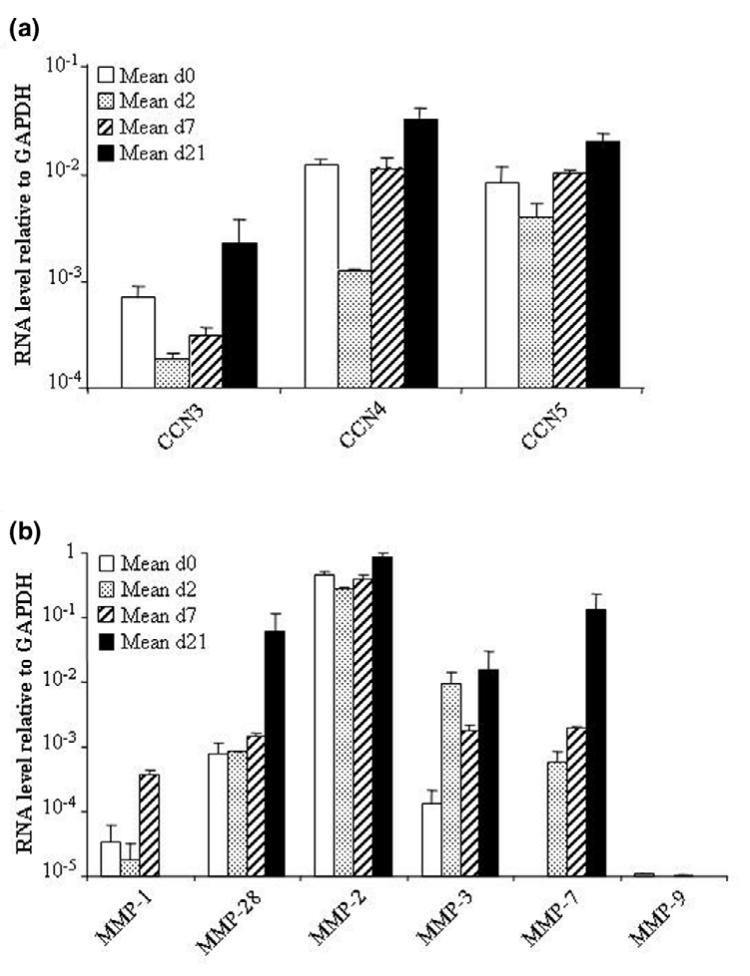
Change in the expression levels of proteins secreted by the chondrocyte-associated extracellular matrix (ECM). The gene-expression profile of mesenchymal stem cells (MSCs; *n *= 2) was analysed by real-time PCR during their differentiation towards chondrocytes in micropellets. **(a) **Time-course expression of the members of the CCN family of genes. **(b) **Time-course expression of various MMPs. CCN, connective tissue growth factor (CTGF); *cef10/cyr61 *and *nov*; GAPDH, glyceraldehyde-3-phosphate dehydrogenase; MMP, metalloprotease; PCR, polymerase chain reaction.

The members of the CCN family are secreted, cysteine-rich, regulatory proteins that interact with growth factors and have important functions in cell proliferation and differentiation in bone and cartilage, in particular [23]. The family includes CCN1/cysteine-rich, angiogenic inducer (CYR)61, CCN2/CTGF, CCN3/nephroblastoma overexpressed (NOV) and the Wnt1-inducible signalling pathway (WISP) proteins (WISP 1 to 3 (CCN4 to 6)). The transcriptional profile of MSCs revealed the expression of CCN1, CCN2, CCN3 and CCN5 (data not shown); these results were validated by quantitative PCR for CCN3, CCN4 and CCN5 (Figure 2a). Our data partly confirm a recent study reporting the mRNA expression of CCN1, CCN2, CCN5 and CCN6 in MSCs, with a decrease in CCN1 and CCN6 expression during the chondrogenic differentiation of MSCs, suggesting that these proteins might be important regulators in the maintenance of the stem-cell phenotype [24]. However, we also observed the expression of CCN3 and CCN4 in MSCs by quantitative PCR and the mRNA levels increased twofold to threefold after chondrogenesis (Figure 2a). To our knowledge, the possibility that CCN3 and CCN4 are expressed in MSCs and expression of CCN3, CCN4 and CCN5 is upregulated after chondrogenesis has not been previously raised. In the study of Schutze and co-workers, the use of semiquantitative RT-PCR instead of real-time PCR might explain the lower degree of sensitivity of the detection [24]. The role of these CCN members in chondrogenesis remains to be elucidated. However, evidence that CCN5 can inhibit the proliferation, invasiveness and motility of vascular smooth muscle cells as a growth-arrest-specific gene has been provided [25]. This suggests that CCN5 might modulate the proliferation of MSCs during the course of the differentiation process.

The timely degradation of the ECM is an important feature of development, morphogenesis and remodelling and mainly mediated by matrix MMPs or matrixins. MMPs are grouped into collagenases, gelatinases, stromelysins, matrilysins, membrane-type MMPs, mainly according to their substrate preference [26]. MMP-1, MMP-2, MMP-3, MMP-7, MMP-9, MMP-12 and MMP-28 are expressed in cartilage [27-29] and, with the exception of MMP-9, we also observed increased levels of expression of the corresponding mRNA after chondrogenic differentiation of MSCs. However, little is known about the expression of these MMPs in MSCs, although the presence of MMP-2 and MMP-3 and absence of MMP-1 and MMP-9 have been reported [30]. We confirmed these data and also showed the expression of MMP-28 and absence of MMP-7 in MSCs and the upregulation during chondrogenesis of all the MMPs tested, except for MMP-9 (Figure 2b). Recently, MMP-9 was shown to be involved in regulating pericellular proteolysis for correct endochondral bone formation during the *in vitro *differentiation pathway and *in vivo *cartilage repair process [31]. Indeed, the absence of MMP-9 in both MSCs and chondrocytes is probably owing to a weak number of cells undergoing terminal differentiation in our culture conditions, as suggested by low (1.75-fold) upregulation of type X collagen in MSCs after differentiation.

### Modulation of the expression of chemokines and their receptors during chondrogenic differentiation

**Figure 3:**
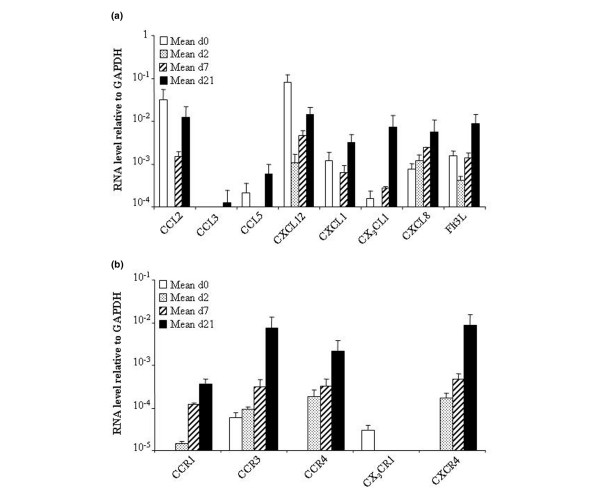
Time-course expression of chemokines and their receptors during chondrogenic differentiation of mesenchymal stem cells (MSCs). The gene-expression profile of MSCs (*n *= 2) was analysed by real-time PCR during their differentiation towards chondrocytes in micropellets. **(a) **Change in the expression levels of various chemokines. **(b) **Change in the expression levels of various chemokine receptors. CCL, chemokine CC motif ligand; CCR, chemokine CC motif receptor; CXCL, chemokine CXC motif ligand; CXCR, chemokine CXC motif receptor; FlT3L, Fms-related tyrosine kinase 3 ligand; GAPDH, glyceraldehyde-3-phosphate dehydrogenase; PCR, polymerase chain reaction.

**Figure 4:**
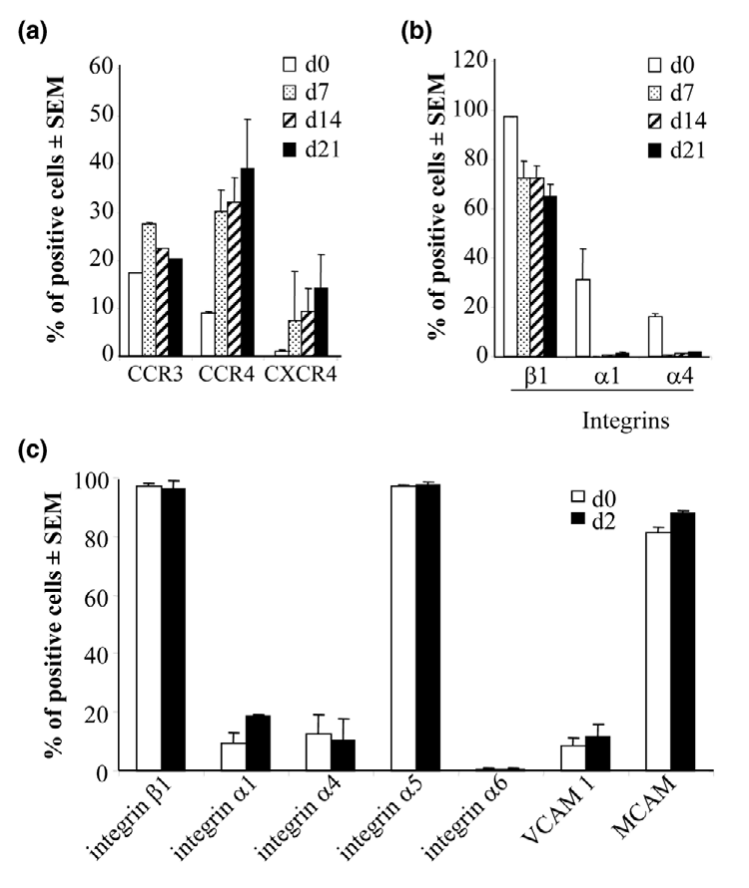
Modulation of the expression of cell-surface proteins on mesenchymal stem cells (MSCs). **(a) **and **(b) **Change in the protein levels of various surface markers during chondrogenic differentiation of MSCs (*n *= 2) in the micropellet. **(c) **Change in the protein levels at the surface of MSCs cultured in monolayer with human BMP-2 for 48 hours. CCR, chemokine CC motif receptor; CXCR, chemokine CXC motif receptor; MCAM, melanoma cell adhesion molecule; SEM, standard error of the mean; VCAM, vascular cell adhesion molecule.

Chemokines are small, heparin-binding proteins that direct the movement of circulating leukocytes to sites of inflammation or injury [32]. Different chemokines have also been demonstrated to influence bone-cell functions, bone-tissue remodelling and stem-cell engraftment [33]. A number of chemokines (chemokine CC motif ligand (CCL)2, CCL4, CCL5, CCL20, CXCL12, CX_3_CL1, CXCL8, CXCL13 and CXCL16) and chemokine receptors (CCR1, CCR7, CCR9, CXCR4, CXCR5, CXCR6 and CX_3_CR1) have been detected in MSCs [34,35]. Here, we confirm the expression of these chemokines by MSCs, when tested and report CXCL1 and Fms-related tyrosine kinase 3 ligand (Flt3L) expression (Figure 3a). One study has already reported the expression of Flt3L in placenta-derived MSCs [36], but, to our knowledge, this is the first report of CXCL1 expression in bone marrow-derived MSCs, at least at the mRNA level. By contrast, most of the chemokine receptors previously described in MSCs were not found, except CX_3_CR1 (Figure 3b). This discrepancy is probably owing to the conditions of cell isolation and/or *in vitro *culture or the fact that only a minority of cells express these receptors [35]. CX_3_CR1 is the only receptor that showed total inhibition of its mRNA during chondrogenesis (from day 2). Interestingly, we detected the expression of CCR3 at the mRNA level, in addition to CCR3, CCR4 and CXCR4 at the protein level (Figure 4a). To our knowledge, the expression of CCR3 was not previously reported in MSCs. Moreover, the levels of all of these receptors increased during chondrogenesis.

**Figure 5:**
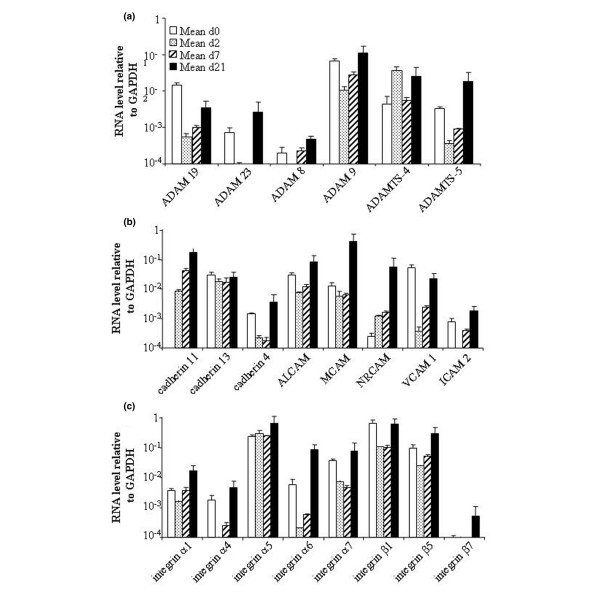
Expression levels of ADAM and adhesion molecules during chondrogenic differentiation of mesenchymal stem cells (MSCs). The gene-expression profile of mesenchymal stem cells (MSCs; *n *= 2) was analysed by real-time PCR during their differentiation towards chondrocytes in micropellets. Change in the expression levels of various ADAM family members **(a)**, several cadherins and CAMs **(b) **and a number of integrins **(c)**. ADAM, A Disintegrin And Metalloproteinase molecule; ALCAM, activated leukocyte CAM; CAM, cell-adhesion molecule; GAPDH, glyceraldehyde-3-phosphate dehydrogenase; ICAM, intercellular CAM; MCAM, melanoma CAM; NRCAM, neuronal CAM; PCR, polymerase chain reaction; ADAMTS, A Disintegrin And Metalloproteinase with thrombospondin type 1 motif; VCAM, vascular cell adhesion molecule.

Chondrocytes are known to express CCR1, CCR2, CCR3, CCR5, CCR6, CXCR1, CXCR2, CXCR3, CXCR4 and CXCR5 [37,38]. Only CCR1, CCR3 and CXCR4 mRNAs were detected in MSC-derived chondrocytes, and we report the expression of CCR4 in these cells (Figure 3b). Interestingly, a huge increase (122- to 2,152-fold) in the levels of these mRNAs was observed after cell differentiation. Their respective cytokines (CCL3, CCL5 and CXCL12) were also expressed in MSC-derived chondrocytes (Figure 3a). Whereas most of the chemokines and their receptors were concomitantly modulated, an inverse correlation between the expression of the CX_3_CR1 and CXCR4 receptors and the expression of their ligands (CX_3_CL1 and CXCL12, respectively) was observed during the course of chondrogenesis. This inversely proportional expression of CXCL12/CXCR4 was recently observed in MSCs cultured on a hyaluronic acid-based scaffold [39]. The authors suggest that the scaffold probably helps to mobilize the internalized receptor, increasing its functional expression and improving engraftment. According to our data, it might also be assumed that the expression of some receptors, notably CXCR4, might reflect progression through chondrogenesis and/or be implicated in the differentiation process. Because interactions between chemokine receptors, syndecans and PGs are known to facilitate the binding of chemokines to their ligands [40], the modulation of the expression of these receptors is probably a crucial event in the migration, attachment or differentiation of MSCs in the specific environment of the joint. In this environment, both immune cells and synoviocytes are other potential sources of chemokines that might influence the migration and homing of MSCs to the cartilage.

### Modulation of the expression of cell-surface markers during chondrogenic differentiation

#### Expression of members of A Disintegrin And Metalloprotease family

ADAM proteins contain a disintegrin and MMP domain, which has the dual function of cleavage/release of cell-surface proteins and remodelling of the ECM [41]. These proteins interact with various partners, such as integrins, syndecans and ECM proteins and are involved in developmental events, including myogenesis, neurogenesis, adipogenesis and morphogenesis. In this study, we report the expression of ADAM8, ADAM9, ADAM19, ADAM23, A Disintegrin-like And Metalloproteinase with thrombospondin type 1 motif (ADAMTS)-4 and ADAM-TS5 in MSCs and their upregulation at the late stage of chondrogenesis, in particular ADAMTS-4 and ADAMTS-5 (Figure 5a). Moreover, we show the presence of ADAM19 and ADAM23, which have not been previously associated with chondrocytes. The involvement of ADAMTS-4 and ADAMTS-5 has already been reported in aggrecan breakdown during endochondral ossification [42]. Expression of the ADAMTS-5 is also increased by Wnt/β-catenin signalling, which was shown to regulate chondrocyte phenotype, maturation and function in a developmentally regulated manner and to be crucial for growth-plate organization and endochondral ossification [43]. These molecules are also implicated in diseases, such as OA and rheumatoid arthritis (RA), in which the ADAMTS-5, expressed in joint tissue, was shown to be the major aggrecanase involved in cartilage destruction [44,45]. Moreover, to our knowledge, this is the first report of the expression of several ADAM proteins, at least at the mRNA level, in MSCs and their upregulation during chondrogenic differentiation. The upregulation of the two ADAMTS molecules at the late stage of chondrogenesis might be related to hypertrophic terminal differentiation. Although the role of the ADAM molecules during the chondrogenic process remains to be elucidated, they might interact with integrins, PGs and ECM proteins to build a microenvironment favouring the appearance of the chondrocytic phenotype.

#### Expression of adhesion molecules

Some adhesion molecules, such as cadherins or CAMs, have important functions in development and tissue morphogenesis, in particular cadherin 11 and N-cadherin, which have a crucial role during MSC condensation [5,46]. The corresponding mRNAs, in addition to mRNAs for cadherins 4 and 13, were expressed in MSCs, and their level of expression was increased on day 21 (Figure 5b). The appearance of transcripts for cadherin 11, as early as day 2, confirms its role during early chondrogenesis and in differentiation. The expression of cadherin 4 and 13 in MSCs has not been previously reported. Because cadherin 4 has been associated with neural retina differentiation [47,48], its role in cartilage development needs further investigation.

Of the adhesion molecules, we present evidence for the expression of all of the CAM molecules tested in MSCs and an increase in their mRNA levels during chondrogenesis, by more than 30-fold for MCAM and neuronal cell adhesion molecule (NRCAM) (Figure 5b). A subset of MSCs is known to express activated leukocyte cell adhesion molecule (ALCAM) (CD166), MCAM (CD146), VCAM1 (CD106) and intercellular cell adhesion molecule (ICAM)2 (CD102) [34], whereas chondrocytes express only ALCAM and VCAM1 [49]. However, NRCAM has not been previously described in MSCs and chondrocytes, and ICAM2 has not been previously described in chondrocytes. NRCAM is involved in the development of the cerebellar system [50] and is a target gene of the β-catenin/lymphoid enhancer binding factor (LEF)-1 pathway in melanoma and colon cancer [51]. Because the Wnt/β-catenin pathway is active in chondrogenesis, it might be speculated that β-catenin induces the upregulation of NRCAM in the micropellet culture. Whether its expression might be involved in cartilage formation, as seen in the cerebellar system, remains to be demonstrated.

#### Expression of members of the integrin family

The integrin family of CAMs are transmembrane glycoproteins, composed of α and β subunits and their combination determines ligand specificity. The patterns of integrin expression determine the adhesive properties of cells by modulating their interactions with specific ECM proteins, suggesting they might be involved in differentiation and migration [52]. MSCs exhibit the expression of integrins α1, α2, α3, α5, α6, αv, β1, β3 and β4 [53]. Here, we confirm these data and show the expression of integrins α4, α7 and β5, whereas integrin β7 is not expressed (Figure 5c). Expression of integrins α1, α4, α5 and β1 was confirmed by FACS analysis (Figure 4b,c). With the exception of integrin α4, which was previously shown to be absent using FACS analysis, integrins α7 and β5 have not been demonstrated to be expressed by MSCs. The lack of detection of the integrin α4 protein by other authors might be attributed to the weak expression of this marker or conditions used for cell isolation/culture.

In chondrocytes, we detected the expression of all of the integrins tested. This confirms previous data reporting expression of integrins α1, α2, α3, α5, α6, α10, αv, β1, β3 and β5 [52]. Integrin α5β1 is the most prominently expressed integrin in chondrocytes and integrin α10β1 is the dominant collagen-binding integrin during mouse cartilage development [52,54]. Although the mRNA levels of the various integrins slightly increase during chondrogenic differentiation (Figure 5c), the protein levels tend to decrease with time (Figure 4b). These data suggest that the subunits of integrins are regulated during the differentiation process. This has already been shown for integrin β1, in addition to a switch from α1 to α3 integrins during chondrogenesis [52]. However, no information is available on the role of integrins α4, α7, β5 and β7 during chondrogenesis. Integrins containing the α4 or α7 subunit bind to RGD-containing components, namely fibronectin and vitronectin or laminin, respectively. Because these components are localized in the cartilaginous ECM, they are probably involved in cellular responses, depending on their interaction with the various integrin receptors expressed by the MSCs.

### Role of bone morphogenetic protein 2 in the modulation of adhesion molecules at the surface of mesenchymal stem cells

Because chondrogenic differentiation was performed by culture in micropellets in the presence of BMP-2, we investigated whether the modulation of expression of some molecules might be induced by BMP-2 and not by the differentiation process. To this aim, we checked the expression of several integrins and two adhesion proteins at the surface of MSC that had been cultured for 2 days in monolayer in proliferative medium with/without BMP-2. We observed the low, but significant, expression of the integrins α1, α4 and α6 and VCAM1 at the protein level, whereas the integrins α5 and β1 and MCAM were highly expressed (Figure 4c). However, no significant increase in integrin expression was measured after stimulation with BMP-2 for 48 hours. These results suggest that BMP-2 does not modulate the expression levels of adhesion molecules, at least for the proteins and time point tested.

## Conclusion

**Figure 6:**
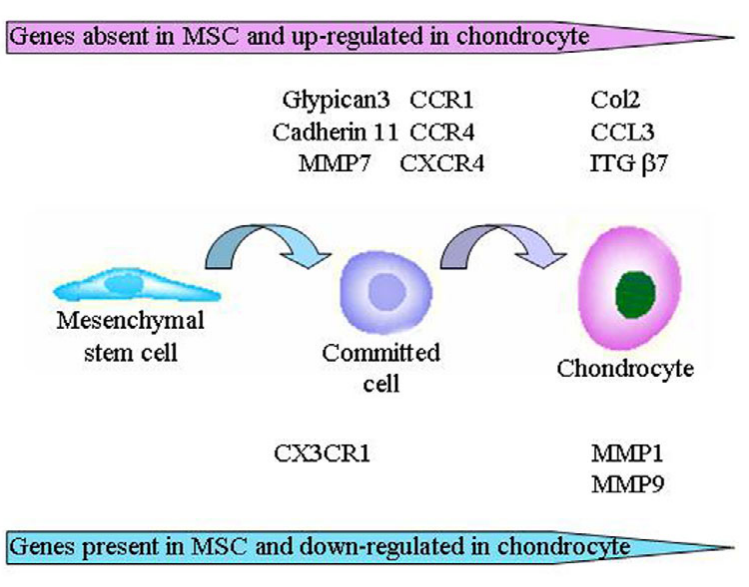
Major changes in the expression of various components of the extracellular matrix (ECM) during the differentiation of MSCs into chondrocytes. CCL, chemokine CC motif ligand; CCR, chemokine CC motif receptor; Col, collagen; CXCR, chemokine CXC motif receptor; ITG, insulin-transferrin-selenic acid; MMP, metalloprotease; MSC, mesenchymal stem cell.

In summary, our study shows that the TLDA could be a useful tool for monitoring the modulation of mRNA profiles during differentiation processes. This assay relies on the use of minor quantities of material (2 ng of total RNA/gene) and quantification of up to 384 genes in the same sample in one experiment. We found that most of the ECM or membrane-associated molecules known to be expressed by MSCs and chondrocytes that are involved in attachment and cell migration, such as PGs, collagens, MMPs, CCN proteins, chemokines and their receptors, ADAM proteins, cadherins and integrins, were reproducibly detected. Indeed, although the study has been performed on few samples, these results are encouraging preliminary data. We suggest that these components are involved in crosstalk between the ECM and the MSCs, which might constitute the direct microenvironment of MSCs within the cartilage tissue, and the chondrogenic process (Figure 6). Moreover, a number of components that were not previously reported to be expressed in MSCs and/or chondrocytes have been identified. Although their expression will have to be confirmed on a statistically relevant number of donors at the protein level, this sets up the basis for a more accurate work on their role in chondrogenesis.

## Abbreviations

α-MEM = α-minimum essential medium; ADAM = A Disintegrin And Metalloproteinase molecule; ADAMTS = A Disintegrin And Metalloproteinase with thrombospondin type 1 motif; ALCAM = Activated leukocyte cell adhesion molecule; b-FGF = basic fibroblast growth factor; BSA = bovine serum albumin; CAM = cell-adhesion molecule; CCL = chemokine CC motif ligand; CCN = CTGF; *cef10/cyr61 *and *nov*; CCR = chemokine CC motif receptor; COMP = cartilage oligomeric matrix protein; Ct = threshold cycle; CTGF = connective tissue growth factor; CXCL = chemokine CXC motif ligand; CXCR = chemokine CXC motif receptor; CYR = cysteine-rich angiogenic inducer; DMEM = Dulbecco's modified Eagle's medium; ECM = extracellular matrix; EDTA = ethylene diamine tetracetic acid; FACS = fluorescence-activated cell sorter; FBS = fetal bovine serum; FlT3L = Fms-related tyrosine kinase 3 ligand; GAPDH = glyceraldehyde-3-phosphate dehydrogenase; hBMP = human bone morphogenetic protein; Hg = Hedgehog; ICAM = intercellular cell adhesion molecule; ITS = insulin-transferrin-selenic acid; LEF = lymphoid enhancer binding factor; mAb = monoclonal antibody; MCAM = melanoma cell adhesion molecules; MMP = metalloprotease; MSC = mesenchymal stem cell; NOV = nephroblastoma overexpressed; NRCAM = neuronal cell adhesion molecule; OA = osteoarthritis; PBS = phosphate-buffered saline; PG = proteoglycan; PTH = parathyroid hormone; RA = rheumatoid arthritis; RT-PCR = reverse transcriptase polymerase chain reaction; SEM = standard error of the mean; TGF = transforming growth factor; TLDA = Taqman^® ^low-density assay; VCAM = vascular cell adhesion molecule; WISP = Wnt1-inducible signalling pathway protein; Wnt = wingless.

## Competing interests

The authors declare that they have no competing interests.

## Authors' contributions

FD performed the majority of the experimental work and participated in the analysis of the data. BD performed the FACS analysis on MSCs. MM performed the FACS analysis on cells isolated from the micropellet. CB participated in the cell-culture work. FA helped in the analysis of the data. PL-P helped in the analysis of the data. FC procured the samples for the isolation of MSCs. PC participated in the analysis of the data. CJ participated in the design of the study and the analysis of the data. DN participated in the design of the study, analysis of data and wrote the manuscript. All authors read and approved the final manuscript. DN and CJ contributed equally.

## Supplementary Material

Additional File 1 An XLS file containing mean 2^-ΔCt ^± SEM results for the complete list of tested transcripts at the different time points. Click here for file
